# Physio-Environmental Sensing and Live Modeling

**DOI:** 10.2196/ijmr.2092

**Published:** 2013-01-30

**Authors:** Filippo Castiglione, Vanessa Diaz, Andrea Gaggioli, Pietro Liò, Claudia Mazzà, Emanuela Merelli, Carel G.M Meskers, Francesco Pappalardo, Rainer von Ammon

**Affiliations:** ^1^Istituto per le Applicazioni del CalcoloNational Research Council of ItalyRomeItaly; ^2^Multiscale Cardiovascular Engineering GroupDepartment of Mechanical EngineeringUniversity College LondonLondonUnited Kingdom; ^3^Department of PsychologyCatholic University of MilanMilanItaly; ^4^Applied Technology for Neuro-Psychology LabIstituto Auxologico ItalianoMilanItaly; ^5^￼Computer LaboratoryDepartment of Computer ScienceUniversity of CambridgeCambridgeUnited Kingdom; ^6^￼Department of Human Movement and Sport SciencesUniversità degli Studi di Roma "Foro Italico"RomeItaly; ^7^School of Science and technologyComputer Science DivisionUniversity of CamerinoCamerinoItaly; ^8^Willem-Alexander Children’s HospitalDepartment of Rehabilitation MedicineLeiden University Medical CenterLeidenNetherlands; ^9^Dipartimento di Scienze del FarmacoUniversità degli Studi di CataniaCataniaItaly; ^10^Center of Information Technology Transfer GmbHRegensburgGermany

**Keywords:** personalized health care, mobile networks, computer models, telediagnosis

## Abstract

In daily life, humans are constantly interacting with their environment. Evidence is emerging that this interaction is a very important modulator of health and well-being, even more so in our rapidly ageing society. Information and communication technology lies at the heart of the human health care revolution. It cannot remain acceptable to use out of date data analysis and predictive algorithms when superior alternatives exist. Communication network speed, high penetration of home broadband, availability of various mobile network options, together with the available detailed biological data for individuals, are producing promising advances in computerized systems that will turn information on human-environment interactions into actual knowledge with the potential to help make medical and lifestyle decisions. We introduced and discussed a key scenario in which hardware and software technologies capable of simultaneously sensing physiological and environmental signals process health care data in real-time to issue alarms, warnings, or simple recommendations to the patient or carers.

## Introduction

### Overview

A proper knowledge of the interaction between human physiology and daily living environmental conditions is essential to establish a connection between an individual’s lifestyle and his/her health status. Understanding these connections will give insight to the effect of pollution on human health.

Most modern prevention or intervention approaches rely deeply on early, accurate, and broad diagnosis, followed by close monitoring of the outcomes. This latter task is carried out by occasional screening and typically produces a series of time dependent snapshots at different levels (eg, biochemical, mechanical, cellular, and molecular). From a biological point of view, every human individual has a different susceptibility to disease. This simple observation has resulted in the concept of personalized medicine and procedures [1]. However, for a personalized treatment to be really effective, accurate individualized information obtained at various levels in a continuous fashion is needed, possibly extending to acquiring a screen of the patients’ home environment.

The aging European Union (EU) population (and of all industrialized countries in general) and the increase in life expectancy are causing a rapid increase of the number of patients with multimorbidity and neurological diseases such as mild cognitive impairment or Alzheimer’s. In this context, the above-described approach in which data are collected in a haphazard way will not suffice anymore. There is an urgent need to shift medical care from institutions to the daily living environment of patients to ensure a continuous follow-up. In addition to this clinical need, there is an economical urgency calling for care distributed differently than the traditional methods. The existing low ratio between care providers and care seekers will become even lower and the growing costs of assistance will soon become unsustainable. Information and communication technology (ICT) tools are already being proposed and studied to provide a solution to these problems, but much more is still expected.

One important aspect is that in general, elderly patients have a more limited capacity to deal with environmental challenges. Moreover, there is increasing evidence that the onset and course of highly prevalent diseases such as stroke, diabetes, and arthrosis are shaped by human environmental interaction (ie, mobility). Assessment and understanding of human-environmental interaction in daily life is therefore of vital importance to design targeted intervention paradigms that aim to optimize conditions such as muscle state, neural plasticity, sensorimotor integration, and internal physiological processes such as insulin metabolism or inflammation. This promotes healthy aging and self-dependency.

### Technological Platforms and Services

The use of technological tools allowing setup of a one-to-many relationship between doctors and patients is potentially an effective strategy for ensuring the necessary quality and intensity of treatment at a sustainable cost [2]. Technological platforms, moreover, allow quantifying the specific progress of a patient, facilitating modulation and customization of treatments, and consequently, a faster recovery. In this respect, a growing number of Web services offer the possibility to track and compare health data. For example, CureTogether [3] allows users to anonymously track various health measures (including symptoms, treatment plans, and medication schedules) and share them with other individuals having the same conditions. Aggregated data can be then analyzed to identify trends and eventually highlight the most effective treatments. Other medical-oriented social networks are appearing, which provide users with tools to track their health status. Collected data, once anonymized, can be used for research purposes, in order to assess, for example, patterns of drug usage or investigate side effects. For instance, PatientsLikeMe [4] is an online platform for patients to share their experience using patient-reported outcomes, find other patients like them, and learn from others’ data to improve their outcomes. The site has gathered a huge quantity of data, with nearly 125,000 members (as of January 2013) spanning a number of different disease communities, including epilepsy, fibromyalgia, and depression.

### Personalization of Treatment and Decisions

Personal medical informatics offers the possibility to store and access data from our daily life and to improve self-knowledge. Insights gained from these measurements can be used, for example, to change life-threatening habits, adopt healthier lifestyle, or make better-informed treatment decisions. From this perspective, the Continua Health Alliance defines personal health system as an “ecosystem of connected technologies, devices, and services” that will enable an “exchange of fitness, health, and wellness information”, in order to “build a community of care” [5]. The final objective of these interoperable personal telehealth solutions is to help health care providers and patients meet “their fitness goals, better manage their chronic diseases, and live independently as they age” [6]. This has been considered in a European-wide context and the EU is currently funding road-mapping exercises for the Digital Patient [7] for example. It is clear that this objective has to be accomplished using a sensitive, respectful, and non-invasive approach, which should not interfere with the patients’ quality of life, and most importantly, should be based on the use of affordable and cost-effective solutions.

Luckily, this objective is within reach. Actually, much of the world now enjoys unprecedented network speed, high penetration of home broadband, and availability of various mobile network options. In this massively interconnected world, capillary information is potentially available to improve medical systems. However, several technical and non-technical issues need to be addressed for the realization of this vision. In particular, the present paper addresses the following key questions: (1) Is it possible to develop new hardware-software technologies capable of simultaneously sensing physiological and environmental signals (eg, temperature, noise), for prolonged times, with little or no invasiveness, and with a level of comfort that ensures wide acceptability? (2) Is it possible to process sensed data in real-time and feed them to integrative models to issue alarms, warnings, or simple recommendations to the subject or to the carers when needed?

### Monitoring Devices

The last decade has witnessed a rapid surge of interest in sensing and monitoring devices for health care and in the use of wearable/wireless devices for a large number of biomedical applications. Body sensors are small pieces of little or non-invasive equipment that measure biophysical parameters (eg, heart beat rate or body temperature). Body sensor technology is growing rapidly (the first international workshop on body sensors was launched in 2004 [8], while the pHealth conference was, as of 2011, already at its 8th edition [9]) and it is becoming available at affordable prices. Similarly, home environments are becoming more and more instrumented, interconnected, and intelligent [10]. The possibility of connecting these data measurement devices with portable communication systems (ie, smartphones) allows for the development of smarter, connected personal health care systems, with the aim to improve diagnosis, treatment, and condition management.

The emergence of body computing is an on-going revolution in hospitals where physicians, medical engineers, and technical personnel deploy handheld mobile devices as clinical computing tools [6,11-15]. The use of tablets and mobile devices coupled with wireless sensor systems (ie, electrocardiography, electroencephalography, electromyography) has the potential to improve quality of care by providing interactive information to institutionalized patients and by facilitating day-hospital and home monitoring activity.

**Figure 1:**
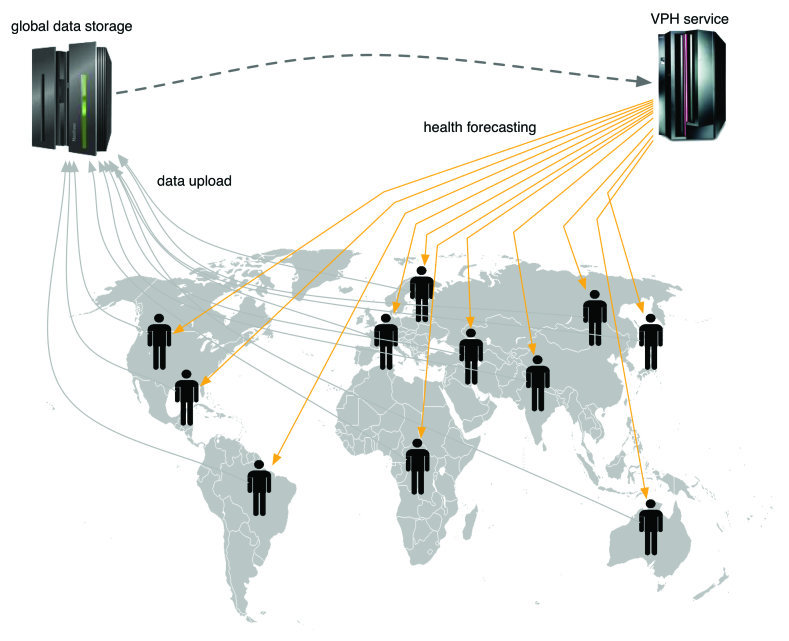
Users upload data via mobile network devices. They get health forecast services through the Web or through ad hoc mobile applications.

However, despite its promises, this approach has found very limited applications. A key barrier is the low integration between mobile and sensor technologies, as different brands use proprietary software platforms for data monitoring and visualization systems. Besides that, a global architecture (or paradigm of data handling at large) for collecting, storing, and using this huge amount of data at a level that can potentially be worldwide, is still missing. A second bottleneck is data analysis. Nowadays, data are stored in databases and mainly analyzed offline, with delayed benefits for everybody, especially for the patients who actually provided the information (this obviously clashes with the personalized medicine concept). There is an urgent need for high frequency data mining, machine learning, and signal processing algorithms to integrate and translate large amounts of data into straightforward readable parameters. These data analysis tools should be based on constantly updated databases, on development of novel statistical methods of causal inference which will be applied to answer causal questions emerging from the data, and on improved pathophysiological models able to interpret data in a predictive, proactive, and possibly automatic, manner. Models predicting the occurrence of a certain event or the emergence of certain behaviour at individual or population level would provide an extraordinary instrument for real-time monitoring. Analytical programs monitoring the sensor data and using rules and logic constraints to describe both the environment and the patient health and to compare against targets, would allow tracking of progress against goals and send alerts when needed (Figure 1). Therefore, health monitoring solutions can become more intuitive, comprehensive, and affordable. Potentially useful applications of these sensor-model integrated systems include (but are not limited to): (1) monitoring patients with chronic diseases (eg, mild cognitive impairments, diabetes, epilepsy, chronic cardiac diseases, progressive renal diseases, and atherosclerosis), (2) monitoring patients that are hospitalized and need frequent probes, (3) monitoring patient’s addiction recovery and long-term drug treatment, (4) monitoring of elderly patients in the daily assessment of generic health conditions. One may raise the point that from the perspective of a developing country, personalized applications might not be economically viable also because prevalent disease pattern differs. However, it is worth to note that the same devices and supportive infrastructure can be tweaked for both clinical and laboratory diagnosis at the health facility level [16]. Indeed, an initiative that is working to address this already exists—the MoDiSe [17].

## Discussion

### Sensors and Models Synergy

As there is a need of flexible ICT tools for supporting software developing in this new application domain, the synergy between computational models and body sensor technology is both imperative and easy to reach. On the one hand, sensors will feed realistic data-driven models for their calibration and validation in areas where this process has traditionally been difficult. On the other hand, predictive models will allow to assess the impact on the population, to optimize the allocation of resources and to devise mitigation and containment interventions to reduce economic and social disruption. Only a perfect intertwining of the two components will make the overall system efficient and efficacious. A crucial feature will be the easiness of use and accessibility to data. In fact, the success of this combined process of data collection, data analysis, and health forecasting will strongly depend on how easy it will be to share the data and to receive information back from the available servers.

The building blocks of such health care distributed system span across areas such as mobile devices, home-based devices, Web-based resources, electronic health records and personal health records. Hence, its development will involve alliances made up from device makers (electronics industry), health care industry, computational modellers (life science researchers) and ultimately also policy makers to institutionalize the integration of this system in the national health care one. It is worth considering the efforts of the Continua Alliance in creating a standardized platform for integrating multiple devices for personalized care [5].

The discussion can be broken down to the following research, technological, conceptual, and societal challenges: (1) developing a whole spectrum of wearable body sensors, (2) developing data communication systems that is secure and allows partial anonymous retrieval to third parties, (3) developing robust storage systems that is extensible and upgradable, (4) developing information systems including computational methods and models exploiting collected such data, (5) developing smart and self-adaptive systems for monitoring the human health.

### Body Sensors

Developing a whole spectrum of wearable sensors capable of measuring cheaply and possibly non-invasively is one of the major challenges, entailing also the development of home-environment sensors that can seamlessly communicate with either public and private wireless or mobile networks to connect to personal data hubs.

**Figure 2:**
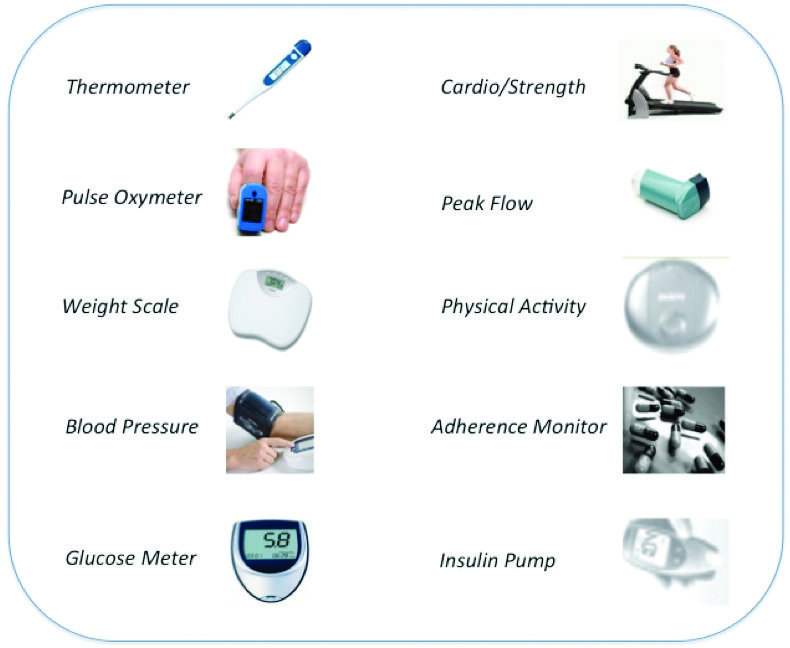
Examples of personal biomedical devices (Source: Continua Health Alliance [4]).

Body sensor research and manufacturing are in continuous and rapid evolution in terms of material, multiphysics, and multiscale physiological integration. Available sensors include electrochemical, optical, and gravimetric sensors and allow for measurements ranging from the whole body scale (inertial devices for movement measurements) to the body structure level (textile-based devices for biological signal monitoring), to the so-called bioelectric diagnostic chips able to scan bodily fluids for various markers of minor illness and disease [18]. See Figure 2 for examples of personal biomedical devices. New and most advanced protein-based sensors are especially interesting as environmental pollutants detectors (ie, sensors based on the folding of proteins, peptides, and DNA when they come into contact with compounds of interest).

Research on implantable in vivo monitoring devices faces problems such as long-term stability and biocompatibility, system integration, sensor miniaturization, low-power sensor interface circuitry design, wireless telemetric links, and on board signal processing. Apart from technological considerations, a lot of effort in this area is devoted to quality and trust of the service/device. In fact, the level of user acceptance strongly depends on how reliable, and hence useful, the proposed technology is, and on how noticeably its output improves his/her quality of life.

The degree of invasive surgery required to implant such devices depends on the type of user. While chronic patients or elder people are more likely to accept technology which promises an improvement in quality of life, healthy individuals might adverse it. This advocates the elderly to be targeted first.

**Figure 3:**
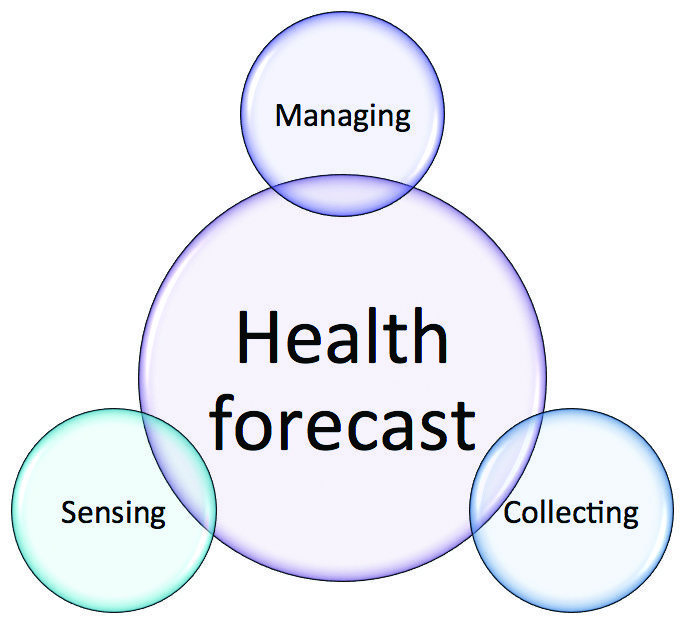
The task of sensing, collecting, and managing the data gains a larger significance when combined together with the possibility to produce new and valuable information on the health status of single individuals or entire populations.

### Data Communication Systems

The data communication network is already available, as it can rely on common data/voice network technology plus the Internet through use of smartphones and tablets. For what concerns the area of wireless sensor networks that could provide interesting solutions for the home and pollution detection sensors, it is necessary to develop wireless protocols, to address the problem of their security, as well as problems related to the performance of large distributed systems, fault tolerance, and anomaly detection.

What needs to be developed is a bulletproof communication workflow that goes from the individual to the storage facility in an anonymous and secure way. Whereas in principle the data could be stored locally on the device and only later uploaded through a secure connection, in general, embedded systems do not have the possibility to store a large amount of data. Hence the development of secure protocols for run-time measurements upload is required.

Imagine a physician’s tool that could evaluate, in minutes or possibly seconds, a wealth of data from connected health devices plus the complete medical history of a patient and all available medical literature (such as medical records, texts, journals, research documents, and even on-going clinical trial results), much of which is unstructured information written in natural language. This application could suggest possible diagnoses complete with documented reasoning or, alternatively, request additional, seemingly unimportant information needed to test hypotheses.

This idea of tracking progress and stay motivated or to monitor chronic conditions and share data with the personal doctor was the original idea behind Google Health [19] that, unfortunately, has been discontinued (end of 2011) because an unexpected insufficient participation to the project. A similar effort (still operational at the time of this writing) is the Microsoft HealthVault [20].

### Storage Systems

The data collection needs to be organized by using taxonomies that are well accepted within and beyond the community of experts dedicated to the development of computational models. For what concerns data standards there exist at least a couple of interesting projects going on. One is the standard for data storage and communication already developed and adopted by both Google Health and Microsoft HealthVault called Continuity of Care Record (CCR) [21] proposed by the American Society for Testing and Materials [22]. This standard is a core data set of the most relevant administrative, demographic, and clinical information facts about a patient's health care, covering one or more health care encounters. It provides a means for one health care practitioner, system, or setting to aggregate all of the pertinent data about a patient and forward it to another practitioner, system, or setting to support the continuity of care. The primary use case for the CCR is to provide a snapshot in time containing the pertinent clinical, demographic, and administrative data for a specific patient [21]. The second is Direct Project launched by the US department of Health and Human Services with the Nationwide Health Information Network initiative in March 2010 [23]. National Health Services (NHS) Interoperability Toolkit [24] in the UK and Healthcare Interoperability Testing and Conformance Harmonisation (HITCH) [25] in the EU are also similar on-going initiatives.

The driving philosophy behind these two efforts is in line with the matter of the present paper. In particular, communication of health information among health care organizations, providers, and patients is traditionally achieved by sending paper through the mail or fax. The development of a standard for data exchange seeks to benefit patients and providers by improving the transport of health information, making it faster, more secure, and less expensive. It will facilitate direct communication patterns with an eye toward approaching unprecedented levels of interoperability.

From an ICT point of view, the development of a general storage system consisting of large data warehouse facilities in charge of providing controlled access to users, does not express a challenge on its own. However, collected data needs to be organized in a strict but also extensible and upgradable manner. This finally comes down to the problem of adopting a standard for names and symbols of biological objects and the use of controlled vocabularies and ontologies to describe repository content [26].

The problem of how to combine data and models in a close synergistic effort to create new information in a way that is both accessible and secure is a stimulating challenge. Besides strictly technical issues, the realization of personal health informatics requires that a number of ethical issues to be addressed. For instance, data from which to derive epidemiological information at the level of geographical regions has an enormous strategic value for industrial sectors as the pharmaceuticals. Data security or integrity is most essential particularly if cloud computing is being considered.

To summarize, data needs to be kept private and secure. It should be shareable with health professionals and downloadable for use elsewhere (also accessible through mobile device). Data should be organized according to standardised ontologies and stored in digital formats that are well defined and already adopted.

### Information Systems

A further challenge concerns the development of information systems (such as computational models embedded in web servers, internet resources, or mobile applications) that are able to exploit collected data and to provide distilled information in forms of predictions. This requires the foundation and development of mathematical and computational methods to achieve prediction on disorder conditions and of diseases spreading in our complex techno-social system. This prompts the development of new, or the adoption of old, large-scale, data-driven mathematical and computational models endowed with a high level of realism. Models enabled by ubiquitous sensors data will allow the forecast of critical events. Moreover, identified modelling needs to motivate the design and implementation of original data-collection schemes. In addition, the setup of computational platforms for disease forecast and data sharing will generate important synergies amongst different research communities and countries.

A critical issue is how to motivate individuals to share their personal data. In fact, as already discussed, the system should rely on the participation of the population to collect real-time information on the distribution of biological parameters or diseases by means of their personal body sensors and smartphone devices (Figure 1). How can individuals be rewarded for spending their time (and money if phone connection is not free) and for sharing personal information with research institutions? In principle, the potential savings that live modelling and continuous monitoring may lead to opens the possibility of applying novel forms of project financing for innovation. In the same way that many public works programs across Europe have been financed through a mix of public and private funds in conjunction with the agreement that the private investors would be entitled to a return on their investment through tolls or the equivalent for a sufficient period of time, cost reducing or controlling eHealth innovation may also attract substantial private investments, if a share of the potential reduction in the cost of treating patients can be passed back to the original private investors in the form an innovation dividend. In a contemporary setting, the value of the saving, from which the original private investors would be entitled a share of, could be derived from the reduction in the average cost of the care of a sufficiently large number of patients with a specific disease within a region that had been selected to trial the innovation in question for a pre-defined duration. This would result in an effective economic incentive for innovation that could attract a wide variety of health care providers, information technology companies, and investments institutions, whilst initially stabilizing (and later on reducing) the costs of health care delivery, management, and innovation.

In the future, scientifically justified health reference costs will be a product of a fully functioning innovative patient- and process-oriented care. This care will be based on live sensors-derived model-guided medicine and on consequent model-guided clinical workflows, spanning the entire health continuum from prevention to diagnosis and treatment to rehabilitation and nursing care. As a result of the expected improvement in early diagnoses and preemptive care, the outcomes of such a system could favourably reflect, in terms of cost, on the contemporary average costs system that was described above. As a consequence, private investors will benefit from a significant return on their innovation investment and health care providers and patients will benefit from lower costs and higher quality of care. In the long run there is even the possibility that the traditional relationship between income (national or individual) and health care expenditure, making health care appear as a luxury good, could be broken and replaced by a relationship in which the core costs of health care delivery and quality are separate from income levels and more closely aligned with innovative solutions to fundamental health care needs. Finally, scientifically justified reference costs and evaluated outcomes-oriented management could replace the black-box (hidden, pragmatic) approaches to health care systems (including diagnostic, therapeutic, systemic, and managerial) and fully exert a role as potential change drivers.

Another critical issue may be the time lag between data collection and individual benefit, which may put extra demands on data collection and processing. The availability of meaningful, directly readable parameters can motivate patients and caregivers, and facilitate online feedback and coaching. This perfectly underlines the necessity to develop powerful data processing algorithms, based on pathophysiological models that are capable of extracting information at far greater speed than is performed nowadays on static databases. An inspiring example was provided by the recent societal and economical phenomena of social networks. These software systems are actually collecting an enormous amount of data without providing any financial reward to individuals. They collect data because people are willing to share information with other people. Note that this is indeed one of the possible reasons for the failure of the GoogleHealth project, as the enthusiasm in sharing personal health data possibly requires the relationship with an institutional partner rather than a software industry. This further suggests that the involvement of institutions in such vision is not optional but rather essential for the successful active support of a critical mass of citizens. HealthSpace, a personal health record platform operated by the NHS that is also suffering from the same disappointing low utilization, provides a suitable anecdote for reflection [27]. This suggests that direct incentives to the patients, citizens, or the population beyond just an institutional support are required.

On the one hand, the system could rely on a kind of social contract whereby motivated individuals have a clear return in term of health assistance. On the other hand, a business model could be adopted to gain from potential market opportunities. The question is whether a system as the one envisioned will prove to live up to user/patient expectations or the whole solution requires a concrete real market opportunity to exploit. Perhaps the answer lies half way between these two extremes in that sensor vendors and communication technology industry can exploit a market opportunity. The data exploitation and health forecast, although curiosity and research driven, will provide enough critical services to boost the interest of a part of the society that is interested and believes in technological advances, especially in the health system.

With respect to data collection, two interconnected points are at the core of the challenge: (1) how to collect the necessary data, and (2) how to ensure that there is no abuse regarding this data. Both questions need to be handled in unison and robust solutions need to be provided if we actually want to employ this technology. Also, this scenario will be markedly influenced by the growing use of electronic patient records that will be spread in the new few years to all clinical activities.

Finally, besides the technical challenges facing the body sensor technology (design, biocompatibility, invasiveness, reliability, energy consumption etc), there are a number of legal, societal, and ethical challenges that need to be addressed.

### Smart and Self-Adaptive Systems

Strictly related to the above subject is the development of smart and self-adaptive systems, that is, intelligent environments for monitoring human health, regulating the uptake of medicaments, and predicting individual emergencies. This includes the development of notice network systems based on overall data able to issue warnings for the population in general.

Smart and self-adaptive systems based on two levels of abstraction, logical and physical, can allow real-time, long-term trend analysis, prediction, prevention, and support of basic daily behavioural and physiological data, building on unobtrusive sensing and advanced reasoning with humans-in-the-loop. The physical level consists of a self-adaptive and self-healing middleware that supports the ensemble of adaptive components and their interactive communication within shared contextual information. The logical level provides tools for automatic reasoning enabling the prediction of spatial/temporal object configurations determining dangerous interactions or physiological damages.

### Impact on Biomedicine

Revolutions in biotechnology and information technology have produced enormous amounts of data and are accelerating the extension of our knowledge of biological systems. These advances are changing the way biomedical research, development, and applications are done. Clinical data complement physiological data, enabling detailed descriptions of various healthy and diseased states, progression, and responses to therapies. It is the availability of data representing various biological states, processes, and their time dependencies that enables the study of biological systems at various levels of organization, from molecule to organism, and even population levels.

Multiple sources of data support a rapidly growing body of biomedical knowledge; however our ability to analyze and interpret these data lags far behind data generation and storage capacity. Mathematical and computational models are increasingly used to help interpret biomedical data produced by high-throughput genomics and proteomics projects. Advanced applications of computer models that enable the simulation of biological processes are used to generate hypotheses and plan experiments. Computational models, appropriately interfaced with biomedical databases, are necessary for rapid access to and sharing of knowledge through data mining and knowledge discovery approaches [28].

Computational biomedicine will provide the possibility of developing not just qualitative but truly quantitative analytical tools, that is, models, on the basis of the data available through the system just described. Information not available today (large cohort studies nowadays include thousands of individuals whereas here we are talking about millions of records) will be available for free.

Large cohorts of data will be available for online consultation and download. Integrative and multi-scale models will benefit from the availability of this large amount of data by using parameter estimation in a statistically meaningful manner. At the same time distribution maps of important parameters will be generated and continuously updated. Through a certain mechanism, the user will be given the opportunity to express his interest on this or that model so to set up a consensus model selection process. Moreover, models should be open for consultation and annotation.

Flexible and user friendly services have many potential positive outcomes. Some examples include simulation of case studies, tests, and validation of specific assumptions on the nature or related diseases, understanding the world-wide distribution of these parameters and disease patterns, ability to hypothesize intervention strategies in cases such as spreading of an infectious disease, and advanced risk modeling.

Notably, these applications are already appearing on the market, for example, a medical device maker company markets a diabetes management solution that combines and analyzes data from a patient’s insulin pump, continuous glucose monitoring device, and blood glucose meter and makes it available to the individual’s doctor. Having a real-time view of blood sugar and the ability to deliver insulin precisely when needed helps diabetics reduce the risks associated with erratic sugar levels.

Along the same lines, it is worth mentioning that the EU project DIAdvisor [29] is developing a prediction-based tool to optimize the therapy of diabetes. In this project, the Ubiquitous Complex Event Processing paradigm recently suggested [30,31] could be applied by using the developed biomarkers and biosensors as event adapters to build a bidirectional event processing communication, eventually into a global event cloud. This would allow a continuous monitoring of the biomarkers and a permanent management of the insulin adjustment, including automatically started processes in the case of specific event patterns (ie, in the case of an emergency).

What was just illustrated is a typical example of a data processing approach that provide direct feedback to the patients about their health status, allows them to be autonomous in the care, reduce the burden to the caregivers and help keep health care budgets within reasonable limits. One of the challenges will be to deal with the increasing complexity of comorbidities typical of the older age, in which it is not feasible anymore to guide interventions based a single parameter. Failure and function of various organ systems have to be taken into account simultaneously (eg, guidance of blood sugar levels with respect to organ damage or cognitive functions). These kinds of choices can only be made based on a thorough knowledge of different clinical phenotypes, on data reflecting the state of different organ systems, and on adequate data-processing algorithms based on pathophysiological models in which disease interactions are taken into account.

### Conclusions

As we learned from a recent study [10], a number of interesting and related surveys have been conducted. One can discover that the rapid adoption of mobile interactive devices has provided a viable gateway for consumers to transmit health data [32]. A survey conducted in North America in 2010 among a sample of 3001 adults ages 18 and older reports that 17% of mobile phone owners (29% of those ages 18-29) use their phones to look up health or medical information. Nine percent of mobile phone owners (15% of those ages 18-29) have smartphone applications that help them track or manage their health [32]. In fact, 10% of all apps downloaded from the Apple iTunes store are related to health care, medical issues, and lifestyle [33]. One example is the Pfizer Mon Krono Santé application, which serves as a memory aid and offers a personal health record for chronic disease sufferers [34]. Gaming devices are viable conduits as well. Bayer’s Didget, for example, is a plug-in for the Nintendo DS gaming system targeting children with diabetes [35]. On other aspects more related to the development of communication protocols for wearable or implantable devices, it is interesting to look at the coordination action of CA-RoboCom [36] that design and describe the Future and Emerging Technologies Flagship initiative, the Robot Companions for Citizens. The Robot Companions for Citizens envisions ecology of sentient machines that will help and assist humans in the broadest possible sense to support and sustain our welfare.

The growing number and increasing maturity of Web-based resources are providing more opportunities for consumer self-service and peer support. Bayer, for instance, offers a comprehensive support program called BETAPLUS for multiple sclerosis patients [37]. In addition to Bayer’s application for the Apple iPhone mobile device that assists with Betaseron injection timing and site reminders, Bayer has also created a robust website with educational tools, forums, and access to solution-trained nurses. Worth mentioning are producers like LUCAS that has developed an innovative and low-cost microscopic appendage to smartphones that is currently being trialled for laboratory diagnosis in Africa [38]. Similar initiatives using microchips or sensors are currently under development by the EU [39].

Clearly, the building blocks are there and gaining traction; but the greater value comes in bringing the components together to provide a step change in diagnosis and treatment both in terms of patient outcomes as well as health care system efficiency. This technology will potentially provide a huge shareable collection of biomedical information worldwide. Devices will be most successful when they provide data that would not otherwise be available because of the measurement frequency required or the need to capture at the right time. This information will be live, meaning that it will be updated constantly and instantaneously. Data crawler and analyzers will extract and produce refined data from the raw data, thus producing interesting distilled information. Mathematical or computational models will use the refined data to make predictions, and medical institutions can use this information to set up health care services such as monitoring systems, warning systems, or aid systems.

Mobile and home-based devices monitor vital signs and activities in real-time and communicate with personal health record services, personal computers, smartphones, caregivers, and health care professionals

Smarter health systems continually analyze information from multiple devices and other sources to derive insights and recommendations for the individual’s health regimes. Here, two examples tackling different levels (or scales) are provided. At the epidemiological/population level, the EU future and emerging technologies (FET) project EPIWORK [40] proposes a multidisciplinary research effort aimed at developing the appropriate framework of tools and knowledge needed for the design of epidemic forecast infrastructures to be used by epidemiologists and public health scientists (also called Internet based surveillance). This pins down the application of the ideas described above, aside from clinical usage, as of potential interest for epidemiological modelling in times of pandemics such as Influence Flu, and epidemics in developing countries and during natural or environmental disasters. At the subject/individual level, another EU project called INTERSTRESS [41], aims at developing a set of personal system tools and services for the collection, classification, and aggregated representation of individual stress patterns.

The virtual physiological human (VPH) vision of the underlying future need focused on monitoring the health status of European citizenship, shares a common view with “The Future of the Internet” [42], that is, to exploit the true unprecedented connecting power of the Internet thanks to mobile and wireless networking and services in order to provide a bridge between market driven research and fundamental research to meet Europe's future needs. Lastly, the proposed vision can have a significant impact on the objectives of the JADE project in the EU [43]. As already mentioned, European citizens are getting older and are increasingly living with chronic diseases. This because although their health condition is better than that of earlier generations, they live longer as a result of advanced medical care and therefore many end acquire chronic conditions and minor disabilities that are well manageable by home care. This has highlighted shared concerns by regional governments about implications for future provision of welfare and health services. This demographic change poses significant challenges to Europe’s society and economy. The JADE project concept is to develop and promote a common research agenda and joint action plan using one of the most promising cluster of ambient intelligence technology applications in everyday life, addressing the need of having independent living services and telecare in an ageing population. They embrace eHealth as an enabler for range activities, for instance, teleconsultations, transfer of records, telehomecare, telehealth, vital sign monitoring, interpersonal communication, remote care, and social support. Among the targeted objectives there are to define new research fields and technologies and to develop actions to improve the cost-effectiveness of research and policy coordination in order to foster transnational scientific cooperation. Finally, another goal of this project is to share and disseminate knowledge awareness on relevant understanding to enhance research, policy effort, and stimulate business actors.

Therefore, we are delighted to report that sensing technology for health is a thriving field of research. It has two immediate and important benefits: (1) the improved understanding of how information and communication technology is revolutionizing human health care, and (2) the investigation of the best conditions for simultaneously sensing physiological and environmental signals. In reaching these targets, it offers the chance to develop computerized systems that will turn this information into actual knowledge with the potential to help making medical and lifestyle decisions. As these questions tackle a range of technological challenges, we believe that combining medical health care meaningfulness, methodological novelties, and the interdisciplinary technological aspects described in this article can lead to ground-breaking applications.

## Abbreviations

CCR: Continuity of Care Record
EU: European Union
FET: future and emerging technologies
HITCH: Healthcare Interoperability Testing and Conformance Harmonisation
ICT: information and communication technology
NHS: National Health Services
VPH: virtual physiological human
